# Adaptive Stereotactic Body Radiation Therapy in the Management of Oligometastatic Uterine Leiomyosarcoma: A Clinical Case Report

**DOI:** 10.7759/cureus.68572

**Published:** 2024-09-03

**Authors:** Maryanne J Lubas, Joseph Panetta, Robert Freeman, Joshua E Meyer

**Affiliations:** 1 Department of Radiation Oncology, Fox Chase Cancer Center, Philadelphia, USA

**Keywords:** stereotactic body radiotherapy (sbrt), cbct-dosimetry, endometrial cancers, adaptive radiation therapy, recurrent leiomyosarcoma

## Abstract

Safe delivery of stereotactic body radiation therapy (SBRT) to large (>5 cm) oligometastatic abdominopelvic tumors can often be challenging, especially in tumors that require a higher biologically effective dose (BED) for tumor control. Adaptive stereotactic body radiation therapy (A-SBRT) involves inter-fraction and real-time replanning while the patient is on the treatment table, potentially allowing for improved dose coverage and greater sparing of critical structures. Our case report illustrates the benefit of CT-based A-SBRT in the treatment and management of an oligometastatic uterine leiomyosarcoma patient with a rapidly enlarging pelvic recurrence. A 60-year-old female presented to the radiation oncology clinic for treatment of an enlarging, right pelvic oligometastatic leiomyosarcoma. She was prescribed 35 Gy in five fractions. Planning prioritized the sparing of nearby small bowels while maximizing coverage of the planning target volume (PTV). On treatment day, two plans were calculated, the initial plan recalculated on the current CBCT (scheduled plan) and a plan reoptimized using current contours (adapted plan), and the more appropriate one was chosen for delivery. The adapted plan was chosen for all five fractions, with the adapted plan offering better small bowel sparing in five fractions and better target coverage in four fractions, delivering a total of 34 Gy to 95% of the PTV while limiting the small bowel to a maximum point dose of 37 Gy. At approximately six months out from treatment, the patient showed continued radiographic response and resolved acute Grade 1 gastrointestinal toxicity. This case study therefore demonstrates the successful treatment of a large oligometastatic abdominopelvic tumor using CT-based A-SBRT and builds on previous experience treating abdominal cases adaptively.

## Introduction

Stereotactic body radiation therapy (SBRT) is an effective local treatment option for patients with oligometastatic cancer with prospective studies demonstrating overall survival and/or progression-free survival or local control benefit following successful ablation of all sites of disease [1-3]. In clinical practice, administering higher, ablative doses to large tumors (>5 cm) in the abdominopelvic region proves technically challenging. Often, these tumors are enmeshed in small or large bowel or may be adjacent to or abutting the kidney, all of which are highly radiosensitive organs [4,5]. As a result, meeting dose constraints for relevant organs-at-risk (OARs) may be difficult and may limit the biologically effective dose (BED) that can be safely delivered to the planning target volume (PTV), with an ideal BED target of >100 Gy.

Moreover, in patients with abdominopelvic leiomyosarcoma, a higher BED is generally required to achieve a durable response, as sarcomas are considered to be more radioresistant tumors with an α/β ratio ranging from two to six [6]. Despite the known radioresistance of these tumors to conventionally fractionated radiation, multiple retrospective and small prospective clinical trials have shown that SBRT may be a beneficial treatment option for patients with unresectable, metastatic sarcoma, leading to improvements in both symptomatic response and local tumor control [7-9].

Recent technological advances in radiation treatment delivery have made SBRT to large abdominopelvic tumors safer and more feasible. Notably, adaptive stereotactic body radiation therapy (A-SBRT), a treatment approach that involves adjusting treatment plans in real-time based on patient daily anatomic changes (as well as tumor size, shape, and position) can be used to potentially minimize OAR dose, while enhancing PTV coverage and minimizing risk of treatment toxicity [10-12]. Although SBRT in the management of leiomyosarcoma has been extensively studied, this clinical case report is the first to our knowledge detailing the use of A-SBRT in the treatment of a patient with oligometastatic uterine leiomyosarcoma with a large (>5 cm) pelvic recurrence.

## Case presentation

**Figure 1:**
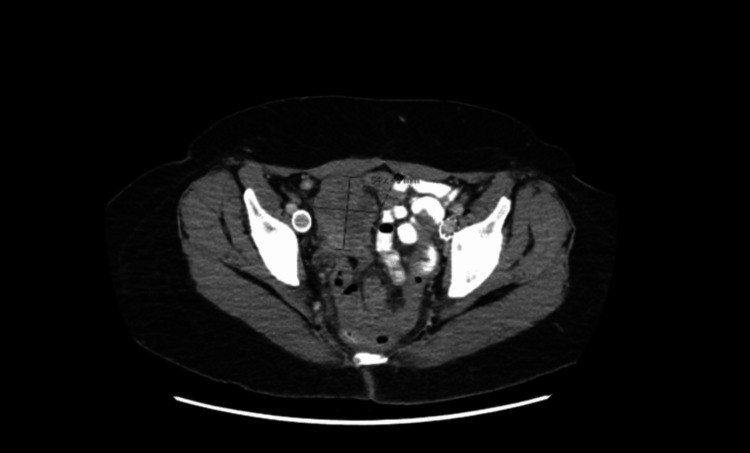
Axial slice CT demonstrating large abdominopelvic mass adjacent to bowel with the right ureteral compression causing hydronephrosis

**Figure 2:**
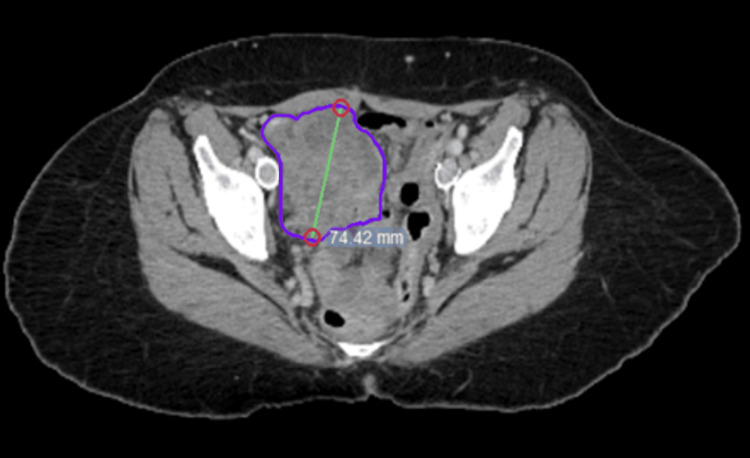
CT simulation scan demonstrating interval growth of the known abdominopelvic tumor compared to the prior scan

A 60-year-old female with a history of recurrent, oligometastatic leiomyosarcoma presented to the radiation oncology clinic for consideration of radiation therapy for an enlarging right pelvic mass. The patient was initially diagnosed with uterine fibroids and underwent a total abdominal hysterectomy and bilateral salpingo-oophorectomy; surgical pathology was consistent with uterine leiomyosarcoma. Approximately six months later, reimaging scans revealed a right pelvic structure measuring 5.0 cm x 4.8 cm, which rapidly grew over the next six weeks. After multiple lines of systemic therapy over several years, the right pelvic mass progressed, eventually measuring 6.1 cm x 4.4 cm as shown in Figure 1. The patient expressed interest in A-SBRT along with planned initiation of pazopanib (an oral, multitarget, tyrosine kinase inhibitor) with medical oncology after completion of radiation therapy. At the time of the CT simulation, scheduled two months later, the pelvic mass measured approximately 8.0 cm x 6.0 cm as shown in Figure 2.

Treatment planning and dosimetry

The patient consented to A-SBRT using the Varian Ethos (Varian Medical Systems, Palo Alto, CA), our CBCT-guided, bore-based adaptive radiotherapy machine. She received 35 Gy in five fractions (BED3=117 cGy, α/β=3) to the PTV, delivered every other day. 

The patient was simulated using a free-breathing CT scan with and without IV contrast and after oral contrast administration; after the fusion of the two scans, the GTV and all OARs (small bowel, large bowel, rectum, bladder, femoral heads, and sacral plexus) were delineated. A 3 mm expansion from the GTV was used to generate the PTV. Because of the overlap of the small bowel and the PTV, a cropped PTV (PTVc), defined as the region of the PTV outside the small bowel, was defined during planning and for all treatment fractions to spare regions of the small bowel that extend within the PTV. An alignment structure consisting of a union of the PTV and the bony pelvis was created to accurately locate the PTV on treatment day. Ring structures around the PTV, defined at 2 mm from the PTV and 2 cm from the PTV, were also created and given dose constraints to produce sharp dose fall-off for any adaptive plans made.

Treatment plans prioritized small bowel sparing followed by coverage of the PTVc and the full PTV. The maximum dose of the target was forced to be greater than 125% in the planning system. Dosimetric goals for the target and dose constraints for OARs used in planning are shown in Table 1.

**Table 1:** Dose criteria for target and OARs applied for the initial plan and all adaptive plans PTVc: cropped planning target volume; OARs: organs-at-risk; D_max_: maximum point dose; fx: fraction

Structure	Criteria
PTV_c_	V_100%_≥95% , 125%≥D_max_≥150%
Small bowel	D_max_≤3800 cGy (760 cGy/fx) , D_30cc_≤2400 cGy (480 cGy/fx)
Rectum	D_max_≤5500 cGy (1100 cGy/fx), D_3.5cc_≤5000 cGy (1000 cGy/fx), D_20cc_≤3800 cGy (760 cGy/fx), D_20%_≤3000 cGy (600 cGy/fx), D_50%_≤2000 cGy (400 cGy/fx)
Bladder	D_max_≤4200 cGy (840 cGy/fx)
Femoral head	V_1990cGy_≤3 cc (760 cGy/fx)
Sacral plexus	D_max_≤3400 cGy (680 cGy/fx), D_5cc_≤3000 cGy(600 cGy/fx)

On treatment day, after the acquisition of the CBCT, the alignment structure was rigidly copied onto the CBCT image and moved until the bony anatomy was aligned. The position and shape of the PTV were then further adjusted by the physician. OARs were automatically created by the artificial intelligence (AI) algorithm of the planning system; these were adjusted by the physician as well. Once all contours were approved, the planning system generated two plans: the initial plan copied and recalculated onto the current anatomy (scheduled plan), and a new plan reoptimized based on the current anatomy (adapted plan). These plans were evaluated and the superior plan was chosen. 

Dosimetric data

Because of the time required to recontour the small and large bowel for each fraction, radiation sessions ranged from 45 min to 69 min, approximately twice the treatment time of non-adaptive SBRT. The patient had no significant issue tolerating lying on the table for the duration of treatment, as she was instructed to take her prescribed pain medications prior to each session.

**Figure 3:**
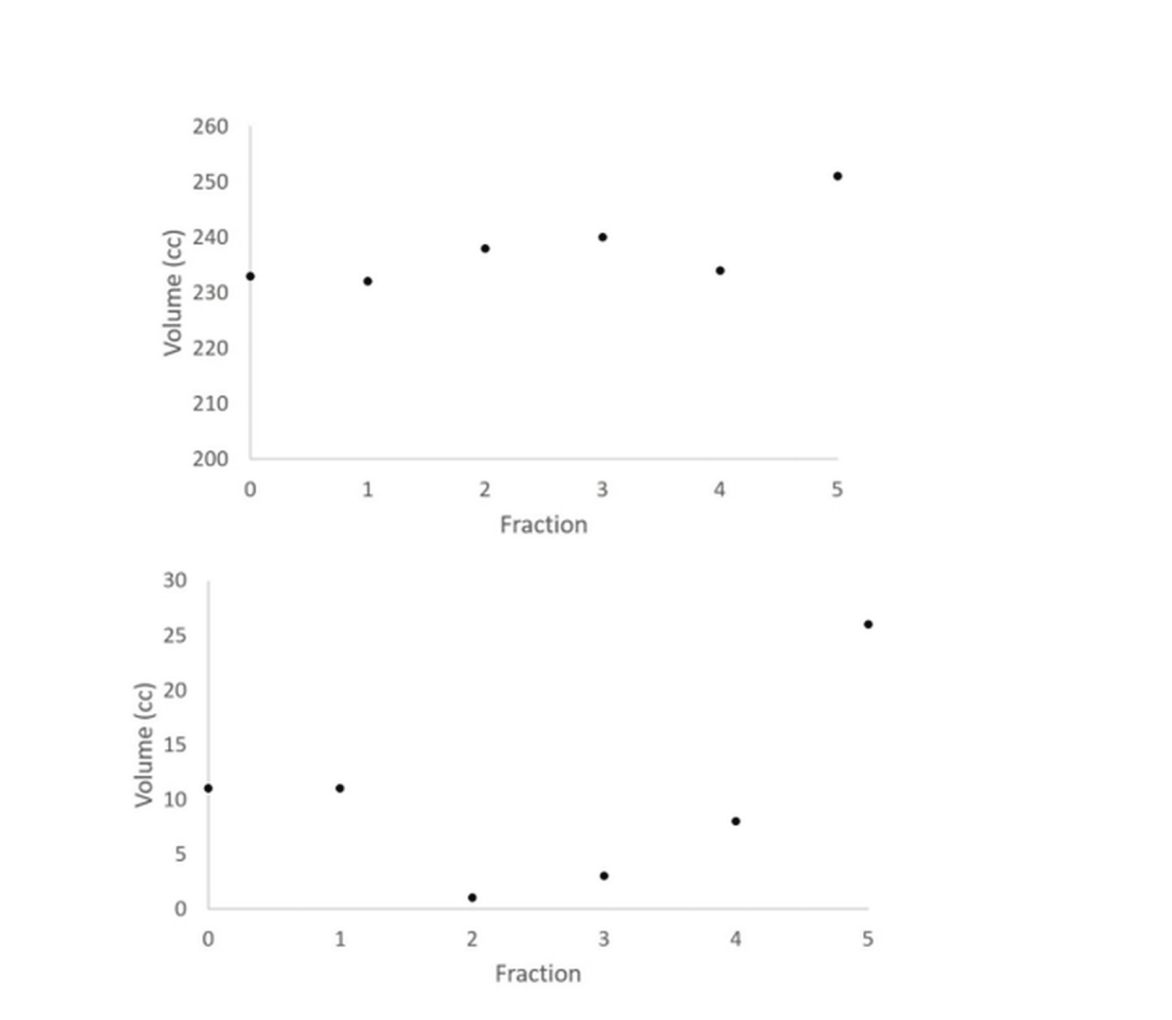
Inter-fractional change in volume of the PTVc (top) and overlap between the PTV and the small bowel (bottom) PTVc: cropped planning target volume; PTV: planning target volume

A dosimetric comparison of adaptive and scheduled fractions is given in Table 2. Target coverage and small bowel sparing were the major determinants of plan quality since other OARs were below acceptable thresholds. The adaptive plan was chosen for all five fractions: in all five fractions, the small bowel dose was lower in the adaptive plan, with an average decrease in small bowel D_max_ and D_30cc_ of 12.0% and 14.5%, respectively. In four fractions, PTV coverage was greater, with an average increase (overall five fractions) in V_100%_ for PTVc and PTV being 1.9% and 1.1%, respectively. Figure 3 shows the change in volume of PTVc as well as that of the tissue that overlapped the PTV and small bowel, demonstrating the difficulty of reliably shielding the small bowel from the dose cloud targeting the PTV without the ability to adapt.

**Table 2:** Comparison of target coverage and small bowel sparing between adaptive and scheduled plans. All increases in V100% and D95 and a percentage decrease in small bowel dose of adaptive plans relative to scheduled plans are also shown PTVc: cropped planning target volume; D_max_: maximum point dose; PTV: planning target volume

Fraction	1	2	3	4	5	Total
PTV_c_ V_100%_ adapted/scheduled (increase by adaptive)	80.7%/91.2% (-10.5%)	94.1%/90.0% (4.1%)	95.0%/92.4% (2.6%)	95.0%/92.2% (2.8%)	95.0%/84.5% (10.5%)	90.0%/92.0% (1.9%)
PTV_c_ D_95 _(cGy) adapted/scheduled (increase by adaptive)	6.2/6.9 (-9.5%)	6.9/6.7 (3.3%)	7.0/6.8 (2.8%)	7.0/6.2 (2.2%)	7.0/6.1 (12.6%)	3260/3410 (4.4%)
PTV V_100%_ adapted/scheduled (increase by adaptive)	84.7%/92.7% (-8.0%)	94.3%/90.2% (4.1%)	94.0%/91.7% (2.3%)	91.9%/90.4% (1.5%)	86.2%/80.5% (5.7%)	90.2%/89.1% (1.1%)
Small bowel D_max_ (cGy) adapted/scheduled (% decrease by adaptive)	729/880 (-17.2%)	737/784 (-6.0%)	736/805 (-17.2%)	752/860 (-13.5%)	745/875 (-14.9%)	3700/4210 (12.2%)
Small bowel D_30cc _(cGy) adapted/scheduled (% decrease by adaptive)	464/611 (-24.1%)	467/526 (-11.2%)	457/525 (-13.0%)	467/540 (-13.5%)	555/621 (-10.6%)	2410/2820 (-14.6%)

Patient outcome

Initial reimaging (three weeks post-treatment) demonstrated a slight increase in the size of the recently treated pelvic mass, with new cystic changes interpreted to be treatment effects. Subsequent repeat imaging (three months post-treatment) showed a decrease in the size of the right pelvic mass from 8.7 cm x 6.0 cm to 6.5 cm x 4.7 cm. Overall, the patient tolerated the treatment well, with acute side effects limited to intermittent abdominal “rumbling” (not painful and did not interfere with her ability to eat or drink) and looser, more frequent stools that resolved without intervention. The patient experienced no acute Grade 2 or higher treatment toxicities including any notable gastrointestinal or genitourinary adverse events. Her increased frequency of urination improved following radiation treatment. At approximately six months post-treatment, the patient shows continued radiographic response and now-resolved acute Grade 1 gastrointestinal toxicity and clinically continues to do well.

## Discussion

Our results demonstrate effective treatment of a large pelvic recurrence of uterine leiomyosarcoma located adjacent to the small bowel. Historically, treatment of large (>5 cm) pelvic lesions with ablative doses of radiation therapy was not feasible due to the radiosensitivity of surrounding OARs and concern for serious acute and late toxicity if dose constraints could not be met. These patients typically received low BED (BED of approximately 50 Gy) and palliative radiation courses for symptomatic lesions [13].

Additionally, given its low alpha/beta ratio, sarcoma tends to be radioresistant in nature. For patients with localized disease treated with either neoadjuvant or adjuvant radiation, local recurrence rates approach 20% with most recurrences occurring in the treatment field [14].

Furthermore, uterine leiomyosarcoma, in particular, proves to be an aggressive histologic subtype, with about 70% of women with stage I and II leiomyosarcoma experiencing a recurrence with an average time to progression of less than one year. These tumors also tend to be somewhat chemoresistant as well, thus further limiting treatment options [15]. Despite the high risk of recurrence, adjuvant systemic therapy has not been found to confer a survival benefit for patients with early-stage disease [16].

As uterine leiomyosarcomas accounts for only 1%-2% of all uterine cancers diagnosed each year (affecting only 0.86 out of 100000 women), conducting large, randomized controlled trials to establish new standard of care treatments is challenging and may take a long time to accrue [16,17]. Thus, new and innovative ways in which to deliver high-dose radiation, while minimizing normal tissue toxicity, are imperative in establishing better disease control in this notoriously difficult-to-treat patient population.

In this case, the adaptive treatment allowed for an increase in fractional dose and a BED3 >100 Gy. The dose regimen for conventional treatment would likely have been limited to 30 Gy in five fractions (BED3=75 Gy). This 14.3% decrease in D_30_ is supported by the total decrease in small bowel D_30_ of 14.6%. In this case, in order to spare small bowel comparably, the average D_95_ of PTVc on a conventional treatment would be 14.6% less the D_95_ of the scheduled plan (Table 2), 28.6 Gy, compared to the of the adapted plan, 34.1 Gy.

While this innovation provides an important opportunity for safe dose escalation for this radioresistant histology, the technology would be expected to allow similar dose escalation in other contexts as well. Therefore, the results of this case report may be applicable to a broad group of patients with oligometastatic disease in the abdomen and pelvis that are surrounded by a small bowel, a frequently dose-limiting structure. The SABR-5 clinical trial evaluated the safety of SBRT in 381 patients treated at up to five metastatic sites. Despite allowing the percentage of the target volume treated with full prescription dose to be as low as 50%, liver and adrenal targets had the highest toxicity rates of any sites: grade 3+ 10.3% and 6.7%, respectively. These patients were not treated with adaptive techniques. Therefore, our case presents a proof of concept demonstrating the possibility of dose escalation, decreased toxicity, or possibly both together [18].

## Conclusions

In this novel case study of a large oligometastatic abdominopelvic tumor, adaptive plans showed superior OAR sparing and target coverage compared to scheduled plans. The patient had good radiographic and clinical response six months post-treatment. Thus, CT-based A-SBRT may be a safe, ablative treatment option for patients with large pelvic tumors, especially those with traditionally radioresistant histology. Longer patient follow-up, prospective studies, and further evaluation regarding optimal BED are needed to determine if A-SBRT can provide durable tumor control in this patient population.
